# Improving the Recycling of Sugar Beet Top–Corncob Waste Through Ensiling with *Lentilactobacillus buchneri* and Cellulase

**DOI:** 10.3390/microorganisms13122761

**Published:** 2025-12-04

**Authors:** Huiling Lin, Jiaxin Li, Junzhao Xu, Baiyila Wu, Zongfu Hu, Huaxin Niu

**Affiliations:** College of Animal Science and Technology, Inner Mongolia Minzu University, Tongliao 028000, China; 19150976657@163.com (H.L.); baiyilaw@imun.edu.cn (B.W.);

**Keywords:** agricultural waste, anaerobic fermentation, microbial community, in vitro fermentation trial

## Abstract

Agricultural wastes such as sugar beet byproducts and corncobs face challenges including high fiber content and low microbe–substrate interaction efficiency during their storage and conversion into animal feed resources. This study evaluated the effects of *Lentilactobacillus buchneri* and cellulase supplementation on fermentation quality, microbial community structure, and the in vitro fermentation rate of mixed silage containing sugar beet tops and corncobs (air-dried). Sugar beet tops and corncobs were mixed at a fresh weight ratio of 9:1 and divided into three treatments—no additives (CK), *Lentilactobacillus buchneri* (LB, 1 × 10^6^ CFU·g^−1^ *Lentilactobacillus buchneri*), *Lentilactobacillus buchneri* and cellulase (LBC, 1 × 10^6^ CFU·g^−1^ *Lentilactobacillus buchneri* and 0.1 g kg^−1^ cellulase)—and subjected to anaerobic fermentation for 60 days. The results showed that LB and LBC treatments reduced the losses of crude protein (CP) and water-soluble carbohydrate (WSC) (*p* < 0.05) and decreased the contents of neutral detergent fiber (NDF) and acid detergent fiber (ADF) (*p* < 0.05). Furthermore, LB and LBC treatments significantly increased the yields of lactic acid (by 31% and 46%, respectively) and acetic acid (by 60% and 78%, respectively) after anaerobic fermentation. Microbial community analysis revealed that *Lactiplantibacillus* (79~85%) was the dominant genus in both LB and LBC treatments, followed by *Levilactobacillus* (9~15%); however, principal coordinate analysis (PcoA) showed significant differences in bacterial communities between the LB and LBC treatment. The LBC treatment significantly enriched *Levilactobacillus*, which exhibited significant positive or negative correlations with multiple fermentation indicators. In addition, in vitro fermentation trial demonstrated that the silage treated with LBC showed higher in vitro dry matter digestibility (IVDMD) and better fermentation characteristics during in vitro fermentation (*p* < 0.05), with significantly increased total volatile fatty acids (TVFA) and butyric acid (BA) contents, and a decreased acetic acid content (*p* < 0.05). During in vitro fermentation, the LBC treatment had higher total gas production, as well as lower methane and carbon dioxide emissions (*p* < 0.05). Under the synergistic effect of *Lentilactobacillus buchneri* and cellulase, the fermentation quality and microbial community of sugar beet top–corncob silage are improved, thereby enhancing in vitro fermentation characteristics and providing insights for the recycling of agricultural wastes.

## 1. Introduction

The increasing demand for meat and other animal-based products is straining the availability of feed, thereby impeding growth within this industry [1]. In order to meet this increasing demand, both livestock production and crop yields have been significantly increased, which has further driven the generation of agricultural waste (AW). China is a major producer of agricultural waste, producing about 5 billion tons annually [2]. However, suboptimal management practices for agricultural waste (e.g., open burning and indiscriminate dumping) generate a significant amount of waste of renewable resources, posing a serious threat to the environment [3]. Consequently, promoting the valorization of agricultural waste can emerge as a pivotal strategy to support the development of the livestock industry. This strategy, in addition to alleviating feed resource scarcity, contributes to the reduction in environmental pollution as well. Agricultural waste is defined as a byproduct that is generated at various stages of the agricultural production process and is primarily composed of crop residues, livestock waste, poultry waste, and agricultural industrial waste [4]. Currently, anaerobic fermentation technology for silage has enabled the conversion of high-moisture fruit/vegetable waste [5], cabbage waste [6,7,8], and sugarcane tops [9] into viable feed resources, and significant research progress has been achieved in this field.

Sugar beet (*Beta vulgaris* L.) is the second-largest sugar crop worldwide, after sugarcane, and is widely cultivated across the world [10]. Sugar beet stems and leaves (tops) are generated as agricultural waste following the harvesting of sugar beets, and when used, yield high levels of crude protein and water-soluble carbohydrates [11]. Therefore, these are classified as significant unconventional feed resources in China. However, the utilization of high-moisture sugar beet tops as a feed source for ruminants is limited by numerous challenges, including perishability, storage issues, and underdeveloped processing technologies. These issues have severely hindered the efficient conversion of sugar beet tops into feed resources and limited their utilization efficiency as feed. According to studies, mixing sugar beet tops along with low-moisture materials (straw, wheat straw) for silage yields better results, notably reducing effluent leakage and decreasing in vitro methane emissions [12,13]. It is, therefore, imperative to study and optimize the silage methodologies to improve the fermentation efficacy and nutrient content. China is a major player in the global corn production landscape and the second-largest global producer of corn [14]. Corncobs (air-dried), a byproduct of corn processing, are available in great amounts but have high fiber content and low palatability, leading to poor intake by ruminants and challenges in their recycling as feed. The combination of sugar beet tops with corncobs (air-dried) for silage can yield better results, potentially resulting in improved feed quality, thereby increasing its utility for ruminants.

Research has explored various silage additives, including lactic acid bacteria (LAB), enzymes, and organic acids, to increase silage quality worldwide. Microbial and enzyme additives play pivotal roles by stimulating beneficial microbiota, inhibiting detrimental microorganisms, accelerating pH reduction, and improving nutritional value. These additive substances effectively improve the fermentation quality, antioxidant capacity, fiber degradation, and in vitro dry matter digestibility [15,16,17]. However, studies have shown that the application of cellulase alone does not achieve ideal yield improvements in fermentation and microbial ecology, whereas using synergistic combinations of cellulase with LAB results in superior efficacy [18,19]. In the study by Yang et al. [18], a compound probiotic containing *Lactiplantibacillus plantarum* and a complex enzyme including cellulase were used for distiller’s grains silage. Du et al. [19] fermented wheat straw with *Lactiplantibacillus plantarum* and cellulase. Both studies demonstrated that the combined application of probiotics and enzymes increased lactic acid content, reduced fiber content, and exhibited superior effects compared to single additions in silages with high moisture and high fiber contents. Furthermore, compared with *Lactiplantibacillus plantarum*, *Lactobacillus buchneri* can additionally enhance acetic acid content, decrease acid detergent fiber (ADF) and neutral detergent fiber (NDF) contents, and improve the microbial community structure during the middle and late fermentation stages [20]. Therefore, we conclude that the combined supplementation of *Lactobacillus buchneri* and cellulase can improve the quality of mixed silage. Furthermore, the synergistic effects of microbes and enzymes can reduce greenhouse gas emissions (notably CH_4_ and CO_2_) during in vitro fermentation by modifying the silage quality and microbial structure [17]. This attribute holds significant potential for mitigating global warming and advancing sustainable livestock production. In this context, our research hypothesis is that fermenting sugar beet tops–corncobs silage via the synergistic interaction of microorganisms and enzymes can improve silage fermentation quality, optimize microbial community structure, enhance its in vitro fermentation characteristics, and mitigate methane and carbon dioxide emissions during in vitro fermentation. Therefore, this study aimed to explore the impact of adding *Lentilactobacillus buchneri* and cellulase on the anaerobic fermentation quality, microbial community composition, and in vitro digestibility of sugar beet top–corncob. The findings of this study are expected to contribute to the scientific strategies for converting high-moisture organic byproducts and fibrous crop residues into valuable feed resources, thereby facilitating waste recycling and supporting circular bioeconomic development in agriculture.

## 2. Materials and Methods

### 2.1. Fermentative Material Preparation

The sugar beet tops selected for this study were the waste of sugar beet roots harvested from farmers’ fields in Chifeng City, Inner Mongolia. The corncobs were air-dried and discarded by farmers in Tongliao City, Inner Mongolia, after which they were collected and ground for use in the experiments in this study. Table 1 shows the nutritional profiles of these unprocessed ingredients, and the values align with those documented in earlier studies [12]. The sugar beet tops were subsequently chopped to 1–2 cm and thoroughly blended with the corncobs at a 9:1 mass ratio, and then divided into three treatment groups (three replicates were arranged for every treatment): no additives with an equal volume of distilled water sprayed instead (CK), sprayed with 1 × 10^6^ CFU·g^−1^ *Lentilactobacillus buchneri* (LB), and sprayed with 1 × 10^6^ CFU·g^−1^ *Lentilactobacillus buchneri* and 0.1 g kg^−1^ cellulase (LBC). 500 g of mixed forage was vacuum-sealed in polyethylene bags, which were then stored at room temperature (22–29 °C) for 60 days to allow for anaerobic fermentation. Afterward, samples were collected and evaluated for silage quality and bacterial community to provide insights into the fermentation process and its outcomes.

The *Lentilactobacillus buchneri* strain used in this study was selected and preserved by our research group, *Lentilactobacillus buchneri* was activated and cultured in de Man, Rogosa, and Sharpe (MRS) broth. Subsequently, the bacterial concentration was determined based on the strain’s growth curve, and the bacterial suspension was diluted with distilled water to a final concentration of ≥1 × 10^6^ CFU·g^−1^ fresh weight (FW). Cellulase (24,000 U·g^−1^) was purchased from Beijing Challenge Agricultural Technology Co., Ltd. (Challenge Group, Beijing, China). Cellulase is dissolved in a diluted bacterial suspension.

### 2.2. Chemical Composition and Fermentation Characteristics of Ensiled Samples

Fresh sugar beet tops and silage samples were collected and dried in an oven at 65 °C for 48 h, after which the dry matter (DM) content was determined. The crude protein (CP) content was determined using a Kjeldahl nitrogen analyzer (K9860, IKEME, Jinan, China) following the analytical protocol specified by the Association of Official Agricultural Chemists [21]. In order to determine the fiber content, neutral detergent fiber (NDF) and acid detergent fiber (ADF) were measured using the filter bag technique, based on Van Soest’s [22] methodology. The content of water-soluble carbohydrates (WSC) was determined through the anthrone-sulfuric acid method, as outlined by Murphy et al. [23].

Next, under sterile conditions, the polyethylene bags were unsealed. Subsequently, 20 g of the fresh sample was homogenized with 180 mL of distilled water, and the resulting mixture was filtered through four layers of gauze using the method described by Song et al. [24]. A pH meter (PHSJ-3F, LEICI, Shanghai, China) was employed to measure the pH. The ammoniacal nitrogen (NH_3_-N) content was quantified according to the phenol-sodium hypochlorite colorimetric method as outlined by Zong et al. [25]. The filtrate obtained after filtration using a 0.22 µm filter membrane [17] was analyzed using high-performance liquid chromatography (GC-14, SHIMADZU, Kyoto, Japan) to determine the concentrations of lactic acid (LA), acetic acid (AA), and butyric acid (BA), following the methods of Ding et al. [26].

### 2.3. Microbial Diversity Analysis

Silage samples were collected and stored in 15 mL centrifuge tubes, which were placed on dry ice for transportation to Meiji Bio for high-throughput DNA sequencing. Genomic DNA was extracted from the microbial communities within these samples using the protocol provided with the E.Z.N.A.^®^ Soil DNA Kit (Omega Bio-tek, Norcross, GA, USA). The extracted DNA samples were analyzed using 1% agarose gel electrophoresis, and the DNA concentration and purity were determined using a NanoDrop2000 spectrophotometer (Thermo Fisher Scientific, Waltham, MA, USA).

The 16S rRNA genes of the bacteria were amplified using the universal bacterial primers 27F (5′-AGRGTTYGATYMTGGCTCAG-3′) and 1492R (5′-RGYTACCTTGTTACGACTT-3′). The primers were subsequently appended with PacBio barcode sequences to differentiate each sample. After PCR amplification, the PCR products were purified with AMPure PB beads (Pacific Biosciences, Menlo Park, CA, USA) and quantified with a Qubit 4.0 (Thermo Fisher Scientific, Waltham, MA, USA). The purified amplicons were then employed for library construction using the SMRTbell Prep Kit 3.0. Sequencing was executed on the PacBio Sequel IIe System (Majorbio Co., Ltd., Shanghai, China). The optimized HiFi reads were clustered into operational taxonomic units (OTUs) using UPARSE version 7.1 at a 97% sequence similarity threshold. For each OTU, the most abundant sequence was identified as the representative sequence. The OTU table was manually filtered to eliminate chloroplast sequences across all the samples. All subsequent bioinformatic analyses of the silage samples were conducted on the Majorbio Cloud Platform (https://cloud.majorbio.com).

### 2.4. In Vitro Fermentation Trial

The produced silages were used for in vitro fermentation trials. Ruminal fluid samples were extracted from four Simmental cows before their morning feeding session, and 300 mL of ruminal fluid was collected from each cow using a stomach tube. The samples were promptly taken to the laboratory and maintained in an insulated container filled with CO_2_ to keep the environment anaerobic. The culture buffer was formulated in accordance with the methods of Menke et al. [27] and subsequently preheated to 39 °C. The collected ruminal fluid was strained through twelve layers of gauze and then combined with the above buffer at a 1:2 ratio (ruminal fluid-buffer, *v*/*v*) to prepare the culture solution. The culture solution was then maintained in a thermostatic water bath at 39 °C. Throughout the entire procedure, a continuous flow of CO_2_ was applied to ensure anaerobic conditions.

The in vitro trial was conducted in line with the method described by Xuan et al. [28]. Briefly, 1 g of each fermentation substrate (DM basis) and 80 mL of the culture solution were thoroughly mixed in a glass fermentation flask (size: 120 mL). The flask was then sealed with a rubber stopper and an aluminum cap, attached to a 300 mL aluminum foil gas collection bag, and placed in a constant-temperature incubator at 39 °C (DQHZ-2001A, CZGY, Changzhou, China) and a shaking frequency of 140 rpm/h for 48 h. In order to ensure experimental repeatability, each treatment was replicated across six glass fermentation flasks. Additionally, three blank flasks containing only the culture solution were included to account for and correct any gas production not attributable to the fermentation substrate.

After 48 h of incubation, an ice-water bath was used to rapidly cool the glass fermentation flasks, ceasing all metabolic activity. Immediately afterward, pH measurements of the culture solution were conducted using a pH meter. The culture solution from each flask was subsequently filtered through a nylon mesh (5 × 10 cm, 200-µm pore size) to separate the residues, which were subsequently washed with distilled water [29] and dried in an oven at 65 °C for 48 h for determining the in vitro dry matter digestibility (IVDMD). Subsequently, 2 mL of metaphosphoric acid were added to 10 mL of filtrate (the filtered culture solution) to preserve samples for the determination of NH_3_-N content and volatile fatty acids (VFA) content. The NH_3_-N content was analyzed using the hypochlorite phenol technique [30]. The content of VFA (AA, PA, BA) were determined using gas chromatography (G2790, Agilent, Santa Clara, CA, USA) as described by Nolan et al. [31]. The total volume of the collected gas was assessed using a glass syringe, and the CH_4_ and CO_2_ compositions were determined using a flame ionization detector-equipped gas chromatograph (TP-2060, Tianpu, Zhengzhou, China) according to the method reported by Kim et al. [32].

### 2.5. Statistical Analysis

To evaluate the effects of different treatments on silage fermentation quality, in vitro fermentation characteristics, IVDMD, and in vitro gas production, one-way analysis of variance (ANOVA) was employed—this method is suitable for comparing differences in a single dependent variable across multiple independent groups. Prior to conducting one-way ANOVA, the Shapiro–Wilk test and Levene’s test were performed to verify data normality and homogeneity of variance, respectively. If the data conformed to a normal distribution (Shapiro–Wilk test, *p* > 0.05) and satisfied the homogeneity of variance assumption (Levene’s test, *p* > 0.05), one-way ANOVA with homogeneous variance was conducted, followed by Duncan’s test for post hoc multiple comparisons to avoid Type I errors (false positives) and provide clear significance results. If the data failed to meet the normality assumption (Shapiro–Wilk test, *p* < 0.05), the Kruskal–Wallis H test—designed for comparing differences among three or more independent sample groups—was used, with subsequent Dunn’s test to prevent false positives caused by multiple comparisons. If the homogeneity of variance assumption was violated (Levene’s test, *p* < 0.05), the Brown-Forsythe test, which is suitable for data with unequal variances, was selected. The threshold for statistical significance was set at *p* < 0.05.

Data analysis of in vitro fermentation characteristics, in vitro digestibility, and in vitro gas production were plotted using GraphPad Prism 9.5 (La Jolla, CA, USA). Different letters denote statistically significant differences. The generation of microbial diversity index boxplots, principal coordinate analysis (PCoA) plots, species composition diagrams, and correlation heatmaps was all performed on the Majorbio Cloud Platform (https://cloud.majorbio.com).

## 3. Results and Discussion

### 3.1. Chemical Composition and Fermentation Characteristics of Silages

The addition of *Lentilactobacillus buchneri* and cellulase significantly enhanced the silage nutritional parameters, with notable effects on CP, WSC, NDF, and ADF (*p* < 0.05) (Table 2).

Previous studies have shown that LAB hydrolyze cellulose to generate pentose sugars or further convert them into organic acids during anaerobic fermentation [33]. In the present study, LBC treatment exhibited the lowest levels of NDF and ADF. This may be due to the addition of cellulase, which disrupts the lignocellulosic structure and accelerates its degradation, thereby promoting the conversion of fiber to WSC [34]. This explains the significantly increased WSC content observed in the LBC treatment. Furthermore, the increase in WSC content observed in the present study may be associated with the decrease in silage pH. A study by Li et al. [34] confirmed that an acidic fermentation environment with a low pH can effectively inhibit the growth of harmful microorganisms, such as *Escherichia coli* and yeast, which also consume WSC in silage as an energy source during their growth. Therefore, the lower pH value of the LBC treatment may inhibit the proliferation of harmful microorganisms, reducing competition with LAB for WSC and allowing more WSC to be retained. The combination of LAB and cellulase improves the degradation of polysaccharides, resulting in the production of more WSC [35]. This also contributed to the significant increase in WSC content observed in the LBC treatment. Proteolysis primarily occurs through microbial and plant enzymatic activity and can be effectively inhibited at pH < 4.6 or through microbial preparations [36]. In the present study, the CP content in both LB and LBC treatments increased significantly, with the LBC treatment showing a significantly higher CP content than the LB treatment. This phenomenon may be due to the degradation of cellulose by cellulase to produce xylose and other monosaccharides, which are subsequently metabolized into microbial proteins [37]. In addition, the increase in CP content is also associated with the dilution effect [38]. The observed elevation of CP content coupled with the reduction in NDF and ADF contents in this study is precisely attributed to this dilution effect. Therefore, incorporating cellulase and *Lentilactobacillus buchneri* during anaerobic fermentation is beneficial for improving the nutritional components of silage.

The addition of *Lentilactobacillus buchneri* and cellulase significantly influenced the pH, NH_3_-N, and organic acid concentrations (*p* < 0.05) in Table 3, thereby effectively enhancing the fermentation quality. LA and pH are vital parameters for silage assessment, with high-quality silage typically exhibiting a pH of < 4.2 [39,40]. Compared to the CK, both LB and LBC treatments resulted in a marked decrease in pH and an increase in LA concentration (*p* < 0.05). The pH range (3.87–3.95) met the quality standard for premium silage. Elevated LA levels may result from the addition of LAB or substrates with high WSC content, which can ferment rapidly to accumulate substantial LA, thereby causing a swift decrease in pH. Compared to the LB treatment, LBC treatment further reduced the pH value and significantly increased the LA content (*p* < 0.05), which may be attributed to the synergistic effect of *Lentilactobacillus buchneri* and cellulase in degrading cellulose to provide ample fermentation substrates [41]. The AA concentration increased significantly in the LB and LBC treatments (*p* < 0.05). *Lentilactobacillus buchneri* (heterofermentative LAB) produces both LA and AA during fermentation [42], which aligns with the findings of this study. Elevated AA suppress undesirable microorganisms (e.g., yeasts and molds), reducing substrate competition and preserving WSC [37,43]. The concurrently observed increase in AA and WSC support this finding. BA was undetectable across all the treatments, demonstrating that co-ensiling beet tops with corncobs generated LA in amounts sufficient to prevent clostridial activity and inhibit spoilage microorganisms [44]. The concentration of NH_3_-N, an indicator of proteolysis, decreased significantly with the addition of *Lentilactobacillus buchneri* and cellulase (*p* < 0.05). This reduction stems from the suppression of proteolytic bacteria (e.g., clostridia) under low-pH conditions [45] and the inactivation of plant proteolytic enzymes at pH < 4.6 [46]. Therefore, incorporating cellulase and *Lentilactobacillus buchneri* during anaerobic fermentation significantly improves silage fermentation quality.

### 3.2. Bacterial Community Sequencing Analysis

**Figure 1:**
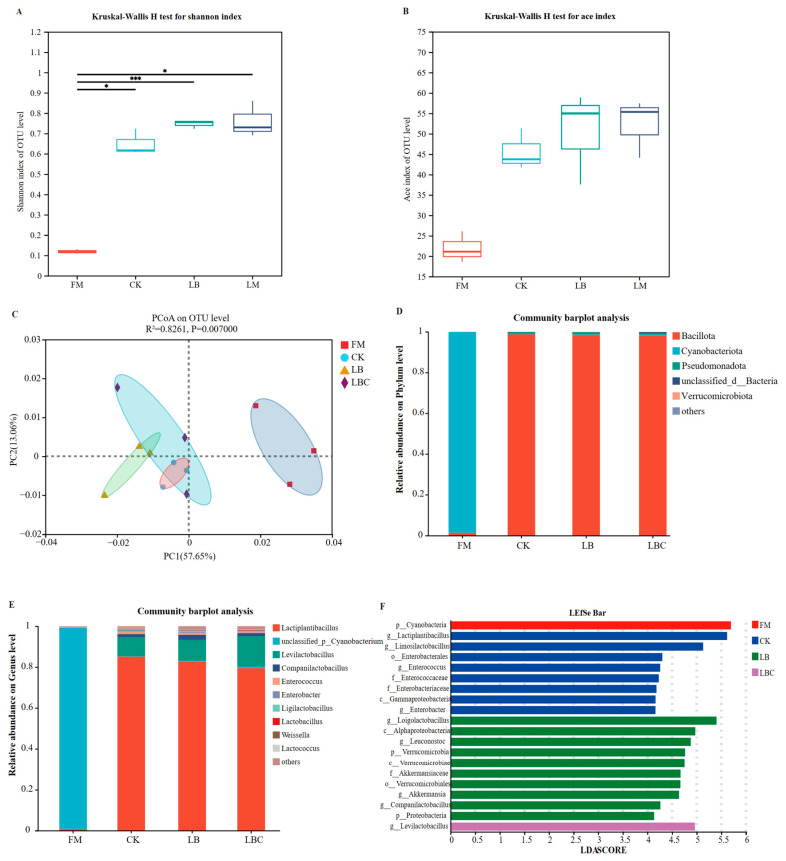
Bacterial community diversity index of sugar beet top–corncob silage (**A**,**B**). Principal coordinate analysis (PCoA) of β diversity in sugar beet top–corncob silage (**C**). Bacterial community composition at the phylum (**D**) and genus (**E**) levels and bacterial community difference analysis (LEfSE) (**F**) of sugar beet top–corncob silage. FM: Fresh sugar beet tops; CK: No additives; LB: *Lentilactobacillus buchneri*; LBC: *Lentilactobacillus buchneri* and cellulase combination treatment. (*p* values are shown as * 0.01 < *p* ≤ 0.05, *** *p* ≤ 0.001).

As shown in Figure 1A,B, the Shannon and Ace indices (reflecting community richness and diversity) both increased after anaerobic fermentation. This study found that supplementation with *Lentilactobacillus buchneri* and cellulase had no significant effect on the bacterial diversity of silage. FM was significantly separated from all silages (CK, LB, LBC) (Figure 1C), confirming the distinct differences in bacterial community structures between FM and silage. CK and LB overlapped with the LBC treatment, indicating a certain similarity in the bacterial communities with the LBC treatment. However, bacterial community separation was also observed between the CK and LB treatments, which is consistent with the findings of He et al. [47].

16S rRNA high-throughput sequencing combined with full-length microbial diversity analysis was employed to characterize the differences in bacterial communities, and LEfSe analysis was performed to identify the taxonomic bacterial communities specific to each treatment (LDA score > 4). The microbial composition at the phylum level is depicted in Figure 1D. FM was dominated by Cyanobacteriota, followed by Bacillota and Pseudomonadota. Notably, the LEfSe results identified Cyanobacteriota as the sole significantly enriched phylum in FM (Figure 1F). Previous studies have demonstrated numerous fresh produce samples all harbor Cyanobacteriota, including mulberry (*Morus alba*) leaves [48], amaranth [49], and alfalfa [50]. In this study, the relative abundance of Cyanobacteriota decreased significantly after anaerobic fermentation, with Bacillota replacing Cyanobacteriota as the dominant phylum (relative abundance > 90%) (Figure 1D) aligning with prior findings [36,51]. This shift is attributed to light deprivation inhibiting photoautotrophic Cyanobacteriota [52,53] and anaerobic, low-pH conditions favoring Bacillota over surface-attached microorganisms [50].

**Figure 2:**
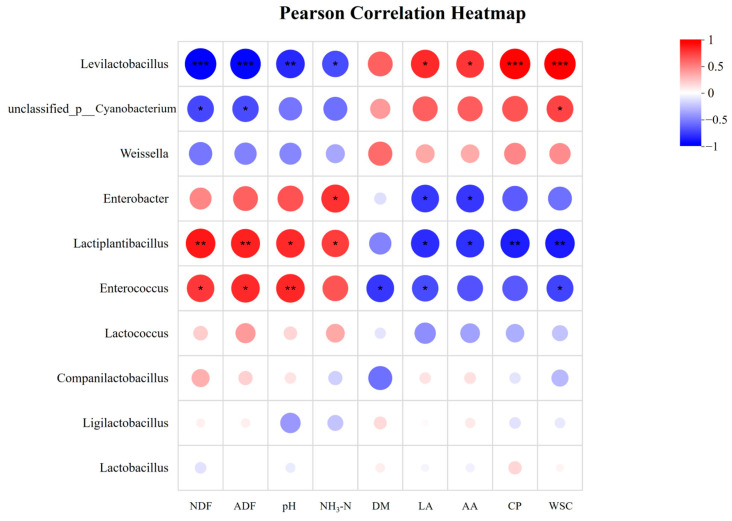
Correlations among the bacterial communities, nutritional components, and fermentation quality of silage. (*p* values are shown as * 0.01 < *p* ≤ 0.05, ** 0.001 < *p* ≤ 0.01, *** *p* ≤ 0.001).

Genus-level analysis (Figure 1C) showed the top three genera in CK, LB, and LBC treatments: *Lactiplantibacillus* (85.14%, 82.76%, 79.58%), *Levilactobacillus* (9.35%, 10.25%, 15.05%), and *Companilactobacillus* (1.50%, 2.46%, 1.37%). After 60 d of anaerobic fermentation, the dominant genus shifted from *unclassified_p__Cyanobacterium* to *Lactiplantibacillus*, which rapidly dominated silage by metabolically inhibiting competitors [54], indicating that the silage had fermented well. *Lactiplantibacillus* (*Lactobacillus plantarum*) inhibits harmful microbes and reduces CP/WSC degradation by producing LA to lower pH, thereby mitigating nutrient loss [55]. The relatively high abundance of *Lactiplantibacillus* in all treatments indicated superior silage quality (relative abundance > 75%). Compared to the CK treatment, the relative abundances decreased in the LB and LBC treatments, with significant enrichment observed in the CK. This may be related to the epiphytic bacterial communities on FM, which inhibit the inoculated strains [56]. Although LB and LBC treatments reduced the relative abundance of *Lactiplantibacillus*, they specifically promoted an increase in the relative abundance of different genera. In the LBC treatment, the relative abundance of *Levilactobacillus* increased, and the genus was enriched in LBC treatment. Similarly, in the LB treatment, the relative abundance of *Companilactobacillus* showed a significant increase, with the genus also being enriched therein (Figure 1E,F). These results suggest that metabolites produced following treatment with LB and LBC, respectively, may promote the growth of *Companilactobacillus* and *Levilactobacillus*. *Lentilactobacillus*, a heterofermentative LAB commonly detected in diverse forage crops and silages, which is correlated with increased LA production and pH reduction [57]. Correlation analysis revealed that *Levilactobacillus* abundance was positively associated with LA concentration and strongly negatively associated with pH (Figure 2). These findings were aligned with the observed increases in LA and decreases in pH. Furthermore, *Levilactobacillus* exhibited extremely significant negative relationships with NDF and ADF, but an extremely significant positive relationship with WSC content (Figure 2). This aligns with the previously noted degradation of fiber by LAB to convert it into sugar sources. Companilactobacillus has been associated with aroma-enhancing components present in fermented foods [58] and has also been detected in silage. Its presence may contribute to improved aromatic flavor, potentially increasing the palatability and forage intake of silage by ruminants. *Lactiplantibacillus*, *Levilactobacillus*, and *Companilactobacillus* are members of the *Lactobacillaceae* family and share similar functional characteristics, probably performing analogous roles during fermentation (e.g., LA production). Therefore, a decline in the relative abundance of *Lactiplantibacillus* may be functionally compensated for, or even exceeded, by increased abundance of *Levilactobacillus* or *Companilactobacillus*. The genus with the highest LDA score in the LB treatment was *Loigolactobacillus* (Figure 1F). The reduction in the abundance of *Loigolactobacillus* is associated with increased acidity [58], which corresponds to the decrease in pH observed in the silage. *Enterococcus* is commonly used as an indicator of animal health, and its excessive abundance is associated with disease; *Enterobacter* and *Lactobacillus* compete for limited nutrients, thereby negatively impacting the silage fermentation quality and nutrient retention [37]. In the present study, the relative abundances of *Enterococcus* and *Enterobacter* in silage were both less than 1%, and these genera were significantly enriched in the CK (Figure 1D), indicating that LB and LBC treatments improved silage quality. Notably, the *Lentilactobacillus buchneri* inoculated in this study did not exhibit enhanced growth and proliferation after ensilage fermentation. The success of inoculated LAB in silage is highly dependent on the competitiveness of the indigenous microbial community [59]. It is likely that the microorganisms attached to the surface of the silage raw materials possess strong adaptability, high competitiveness, and large quantities, thereby inhibiting the growth of *Lentilactobacillus buchneri*. Additionally, *Lentilactobacillus buchneri* has lower acid tolerance compared to *Lactiplantibacillus* (a genus of homofermentative LAB) [60], resulting in the rapid decrease in pH value after ensilage fermentation, which further suppresses the growth activity of *Lentilactobacillus buchneri*.

The associations between silage fermentation parameters and the top 10 bacterial genera were evaluated using Pearson correlation analysis, and the results are illustrated in Figure 2. Both direct and indirect associations were noted between microbial composition and silage nutritional components and fermentation quality. *Levilactobacillus* showed a significant negative correlation with NH_3_-N and positive correlations with WSC and CP, implying that it may reduce CP degradation to enhance nutrient preservation. *Levilactobacillus* plays a key role in the rapid acidification of the silage environment and inhibition of microbial activity during the later fermentation stages [49]. Notably, *Lactiplantibacillus* (*Lactobacillaceae*) exhibited a significant positive correlation with pH and a significant negative correlation with the LA. This contrasts with *Levilactobacillus*, suggesting that *Levilactobacillus* may exert similar roles during anaerobic fermentation, thereby attenuating the relevant characteristics of *Lactiplantibacillus*. *Enterobacter* was strongly positively correlated with NH_3_-N and negatively correlated with the LA. This confirms that higher *Enterobacter* abundance drives competition between harmful microorganisms and LAB, which consume silage nutrients and lower organic acid production.

### 3.3. In Vitro Fermentation Trial of Silages

**Figure 3:**
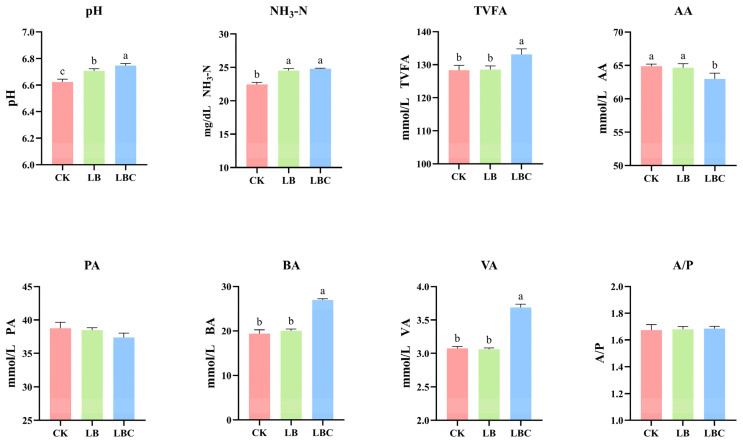
Effect of *Lentilactobacillus buchneri* and cellulase on the in vitro fermentation of sugar beet top–corncob silage. CK: No additives; LB: *Lentilactobacillus buchneri*; LBC: Combination treatment with *Lentilactobacillus buchneri* and cellulase. TVFA, Total volatile fatty acid; AA, Acetic acid; PA, Propionic acid; BA, Butyric acid; VA, Valeric acid; A/P, Acetic acid to propionic acid ratio. Different lowercase letters indicate significant differences (*p* < 0.05).

Figure 3 shows the rumen fermentation characteristics of silage after 48 h. Rumen pH is a critical indicator of fermentation status, significantly influencing microbial activity. Both excessively high and low pH values will reduce rumen fermentation performance [61]; a pH of around 6.5 results in optimal rumen fermentation [62]. In this study, the LB and LBC treatments resulted in ruminal pH significantly elevated (*p* < 0.05), although remained within normal physiological limits (6.62–6.75). The NH_3_-N concentrations in the LBC and LB treatments significantly exceeded those observed in the CK treatment (*p* < 0.05), which is consistent with the findings reported by Li et al. [17]. As the primary nitrogen source for rumen microbes, NH_3_-N is derived from the degradation of feed proteins/non-protein nitrogenous [63,64]. LBC and LB treatments likely promoted the degradation of nitrogenous substances such as rumen crude protein, increasing NH_3_-N production. Concurrently, proteolysis is accompanied by the formation of alkaline compounds, which elevates rumen pH [65], and explains the increased pH observed in the LB and LBC treatments. Volatile fatty acids (VFAs), which provide > 80% of ruminant energy [66], reflect in vitro fermentation efficacy. Total volatile fatty acid (TVFA) production increased significantly in the LBC treatment (*p* < 0.05) in this study, suggesting that *Lentilactobacillus buchneri*-cellulase synergism improved the degradation of sugar beet top–corncob mixed silage. This enhancement likely stems from the disruption of plant cell walls, facilitating microbial access to nutrients and increasing the IVDMD and VFA yield. In the rumen, rumen bacteria can convert AA into BA via acetyl-CoA or butyryl-CoA transferase [9,67]. The results revealed that in the LBC treatment, AA levels significantly increased, whereas BA levels notably decreased, which may be attributed to the conversion of AA to BA.

**Figure 4:**
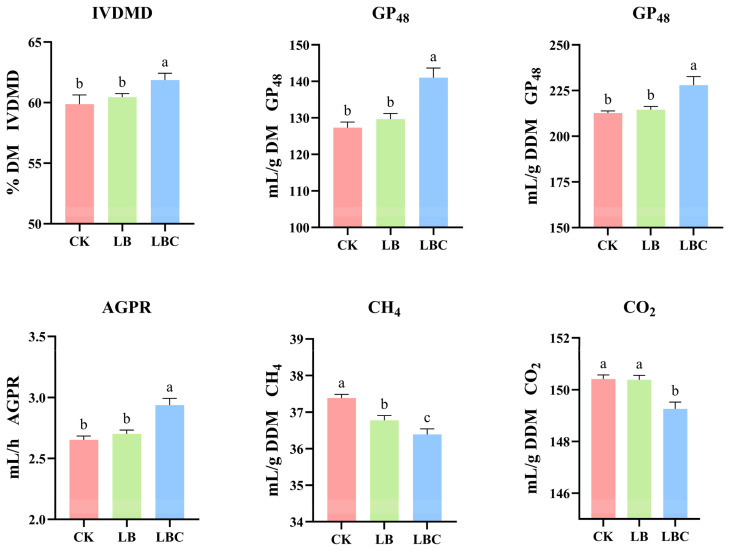
Effects of *Lentilactobacillus buchneri* and cellulase on the IVDMD, gas production, CH_4_, and CO_2_ production in sugar beet tops–corncobs silage. CK: No additives; LB: *Lentilactobacillus buchneri*; LBC: *Lentilactobacillus buchneri* and cellulase combination treatment. IVDMD, in vitro dry matter degradability; DM, Dry matter; DDM, Digestible dry matter; GP_48_, Cumulative gas production at 48 h; AGPR, Average gas production rate at the time when half of A occurred. Different lowercase letters indicate significant differences (*p* < 0.05).

The in vitro fermentation was simulated the kinetic processes of rumen feed fermentation and evaluated the degradation efficiency of rumen microorganisms on different feeds [68]. The values of the parameters, including IVDMD, GP, and greenhouse gas emissions (CH_4_, CO_2_), are presented in Figure 4 and Table A1. GP is highly correlated with in vitro degradation parameters [54]. IVDMD reflects substrate digestibility by the rumen microbiota, whilst GP effectively indicates the microbial substrate utilization efficiency and the nutritional value of the feed. In this study, inoculating with *Lentilactobacillus* *buchneri* has no impact on the degradability characteristics of the rumen [69]. The synergistic effect of *Lentilactobacillus buchneri* and cellulase significantly increased IVDMD and GP (*p* < 0.05), increasing ruminal degradation. This finding was aligned with the reports of improved fiber digestibility following LAB-enzyme treatment [41]. Cellulase hydrolyzes plant cell walls, reducing the NDF and ADF contents and thereby improving digestibility. Elevated ruminal pH in LBC may further promote fibrolytic bacterial activity [70], which explains the increased IVDMD. The degradation of protein, carbohydrates, and carbon in the feed, which is accomplished by microbial activity, is the primary process responsible for total gas production in the rumen [71]. Compared to those in the CK and LB treatments, the silage in the LBC treatment presented significantly greater concentrations of CP and WSC (*p* < 0.05), which led to a marked increase in GP. However, no significant variation in gas production was observed between the LB and CK treatments, which may be associated with the composition of the silage cell walls.

Enteric CH_4_ from ruminants constitutes ~30% of the total anthropogenic methane emission [72] and has a 26-fold greater global warming potential than CO_2_. About 88% of enteric CH_4_ is derived directly from ruminal fermentation, accounting for 6–12% of ruminants total energy intake (GEI) or 8–14% of their digestible energy intake (DEI) [73,74]. Compared to the CK and LB treatments, LBC treatment resulted in a marked decrease in CH_4_ and CO_2_ emissions (*p* < 0.05), which is consistent with the findings reported by Li et al. [17]. A reduced CH_4_ indicates improved energy utilization efficiency [75]. Hydrogen gas is released by the rumen during acetic acid synthesis [76]. Correspondingly, the volume of hydrogen generated in the LBC treatment group decreased as the AA concentration decreased. As a result, the methane and carbon dioxide outputs through the methyl compound-hydrogen reduction pathway were likewise decreased.

From the perspectives of agricultural production and livestock productivity, the synergistic addition of *Lentilactobacillus buchneri* and cellulase significantly improves the fermentation quality of silage produced from agricultural wastes (sugar beet tops and corn cobs), thereby enhancing in vitro fermentation efficiency and directly translating to increased feed intake and digestibility in ruminants. Furthermore, the reduction in greenhouse gas emissions alleviates the environmental pressure associated with livestock production, aligning with global low-carbon agriculture goals. This technology enables the recycling of agricultural waste, reducing reliance on conventional feed resources while effectively lowering production costs. Overall, these findings provide a feasible and sustainable solution for ruminant feeding systems.

## 4. Conclusions

This study showed combining *Lentilactobacillus buchneri* and cellulase as a silage additive modulated the microbial community (promoting beneficial bacteria such as *Levilactobacillus* and *Companilactobacillus*), enhanced acetic acid, lactic acid production, neutral detergent fiber and acid detergent fiber degradation, thereby reducing nutrient and protein loss during anaerobic co-fermentation of sugar beet tops and corncobs. Additionally, the treated silage improved in vitro dry matter digestibility, total volatile fatty acid and gas production, while lowering CH_4_ and CO_2_ emissions in vitro fermentation. These findings provide important information for high-moisture sugar beet tops silage and corncobs agricultural waste for ruminant high value utilization.

## Figures and Tables

**Table 1:** Chemical characteristics of sugar beet tops and corncobs before ensiling.

Items	Sugar Beet Tops	Corncobs (Air-Dried)	Sugar Beet Top–Corncob
DM (g/kg FM)	168.17 ± 4.12	907.40 ± 4.81	243.61 ± 3.63
CP (g/kg DM)	161.03 ± 7.51	46.73 ± 1.01	146.23 ± 2.15
NDF (g/kg DM)	226.21 ± 5.31	629.73 ± 10.29	442.13 ± 3.19
ADF (g/kg DM)	159.69 ± 6.80	339.52 ± 9.31	261.47 ± 2.90
WSC (g/kg DM)	193.54 ± 8.38	96.92 ± 7.13	83.31 ± 1.08

FM, fresh matter; DM, dry matter; CP, crude protein; NDF, neutral detergent fiber; ADF, acid detergent fiber; WSC, water-soluble carbohydrate. Mean ± standard error.

**Table 2:** Effect of *Lentilactobacillus buchneri* and cellulase on the nutritional composition of sugar beet top–corncob silage.

Items	Treatments			*p*-Value
	CK	LB	LBC	
DM (g/kg FM)	230.50 ± 9.47	229.57 ± 5.48	238.40 ± 4.77	0.298
CP (g/kg DM)	108.43 ± 2.46 ^c^	112.50 ± 0.95 ^b^	120.03 ± 0.98 ^a^	<0.001
NDF (g/kg DM)	409.00 ± 8.20 ^a^	394.80 ± 1.20 ^b^	338.83 ± 7.27 ^c^	<0.001
ADF (g/kg DM)	233.43 ± 10.32 ^a^	212.77 ± 7.27 ^b^	182.63 ± 5.06 ^c^	<0.001
WSC (g/kg DM)	21.83 ± 0.55 ^c^	24.17 ± 0.25 ^b^	30.67 ± 0.50 ^a^	<0.001

CK: No additives; LB: *Lentilactobacillus buchneri*; LBC: Combined treatment with *Lentilactobacillus buchneri* and cellulase. FM, Fresh matter; DM, Dry matter; CP, Crude protein; NDF, Neutral detergent fiber; ADF, Acid detergent fiber; WSC, Water-soluble carbohydrate. Different lowercase letters indicate significant differences (*p* < 0.05); the same applies below.

**Table 3:** Effect of *Lentilactobacillus buchneri* and cellulase on the fermentation characteristics of sugar beet top–corncob silage.

Items	Treatments			*p*-Value
	CK	LB	LBC	
pH	3.95 ± 0.03 ^a^	3.89 ± 0.02 ^b^	3.85 ± 0.02 ^c^	0.004
NH_3_-N (%)	1.55 ± 0.04 ^a^	1.08 ± 0.02 ^b^	1.01 ± 0.03 ^c^	<0.001
LA (g/kg DM)	43.94 ± 1.93 ^c^	57.68 ± 2.63 ^b^	64.04 ± 0.97 ^a^	<0.001
AA (g/kg DM)	6.81 ± 0.19 ^c^	10.90 ± 0.53 ^b^	12.13 ± 0.33 ^a^	<0.001
BA (g/kg DM)	ND	ND	ND	

CK: No additives; LB: *Lentilactobacillus buchneri*; LBC: *Lentilactobacillus buchneri* and cellulase combination treatment. LA, Lactic acid; AA, Acetic acid; BA, Butyric acid; NH_3_-N, Ammonia nitrogen; ND, Not detected. Different lowercase letters indicate significant differences (*p* < 0.05).

## Data Availability

The metagenomic sequencing raw data for our samples have been deposited in the NCBI Sequence Read Archive (SRA) under accession number: PRJNA1321521.

## Abbreviations

CK: No additives; LB: Lentilactobacillus buchneri; LBC: Combination treatment with Lentilactobacillus buchneri and cellulase. DM, Dry matter; CP, crude protein; NDF, neutral detergent fiber; ADF, acid detergent fiber; WSC, water-soluble carbohydrate; NH_3_-N, Ammonia nitrogen; LA, Lactic acid; AA, Acetic acid; PA, Propionic acid; BA, Butyric acid; IBA, Isobutyric acid; VA, Valeric acid; TVFA, Total volatile fatty acid; A/P, Acetic acid to propionic acid ratio.

## Appendix A

**Table A1:** Effect of *Lentilactobacillus buchneri* and cellulase on IVDMD, gas production, CH_4_, and CO_2_ production of sugar beet tops–corncobs silage.

Items	Treatments			*p*-Value
	CK	LB	LBC	
IVDMD (% DM)	59.86 ± 0.77 ^b^	60.45 ± 0.30 ^b^	61.85 ± 0.57 ^a^	0.014
GP_48_ (mL/g DM)	127.33 ± 1.53 ^b^	129.67 ± 1.53 ^b^	141.00 ± 2.00 ^a^	<0.001
GP_48_ (mL/g DDM)	212.71 ± 1.19 ^b^	214.50 ± 1.76 ^b^	227.99 ± 4.29 ^a^	<0.001
AGPR (mL/h)	2.65 ± 0.03 ^b^	2.70 ± 0.03 ^b^	2.94 ± 0.04 ^a^	<0.001
CH_4_ (mL/g DDM)	37.39 ± 0.10^a^	36.78 ± 0.13 ^b^	36.39 ± 0.11 ^c^	<0.001
CO_2_ (mL/g DDM)	150.41 ± 0.16 ^a^	150.24 ± 0.16 ^a^	149.19 ± 0.22 ^b^	<0.001

CK: no additives; LB: *Lentilactobacillus buchneri*; LBC: a combined treatment of *Lentilactobacillus buchneri* and cellulase. IVDMD, in vitro dry matter degradability; DM, dry matter; DDM, digestible dry matter; GP_48_, cumulative gas production at 48 h; AGPR, the average gas production rate at the time when half of A occurred.
